# Neck circumference as a screening measure for identifying NAFLD among a group of academic employees in Bangkok, Thailand

**DOI:** 10.1371/journal.pone.0263826

**Published:** 2022-02-17

**Authors:** Sapwarobol Suwimol, Tangkijvanich Pisit, Avihingsanon Anchalee, Kongruttanachok Narisorn, Jantarapakde Jureeporn, Jiamjarasrangsi Wiroj

**Affiliations:** 1 Medical Food Research Group, Nutrition and Dietetics Department, Faculty of Allied Health Sciences, Chulalongkorn University, Bangkok, Thailand; 2 Center of Excellence in Hepatitis and Liver Cancer, Faculty of Medicine, Chulalongkorn University, Bangkok, Thailand; 3 HIV-NAT, Thai Red Cross AIDS Research Centre, Bangkok, Thailand; 4 Department of Laboratory Medicine, Faculty of Medicine, Chulalongkorn University, Bangkok, Thailand; 5 Department of Preventive and Social Medicine, Faculty of Medicine, Chulalongkorn University, Bangkok, Thailand; Auburn University, UNITED STATES

## Abstract

Neck circumference (NC) reflects the fat deposition in upper body and has potential to be used as a predictor of Non-Alcoholic Fatty Liver Disease (NAFLD). Our objectives were to examine the association of NC with NAFLD prevalence, and to determine the optimal cut-off of NC in identifying the presence of NAFLD among the employees of an academic institution in Bangkok, Thailand. In this cross-sectional study, 635 employees of an academic institution underwent anthropometric measurement and transient elastography following an overnight fast. NAFLD was defined as a CAP value >238 dB.m-1. The NAFLD prevalence in men and women were 66.17% and 46.22%, respectively. The mean NCs for men and women with NAFLD were higher (38.53±0.31 cm and 35.83±0.48 cm, respectively) than those without NAFLD (33.58±0.24 and 31.098±0.14 cm, respectively) (p<0.001). Metabolic markers including age, weight, BMI, NC, WC, WHR, FBS, triglycerides were significantly higher, HDL was significantly lower among participants with NAFLD compared to those without NAFLD (p<0.05). NC was independently associated with NAFLD among women with OR (95%CI) of 1.17 (1.05, 1.32). The optimal cut-offs of NC to predict NAFLD were 37.07 cm (sensitivity: 70.50%; specificity: 68.90%) and 32.07 cm (sensitivity: 70.70%; specificity: 62.10%), respectively for men and women. NC significantly correlated with NAFLD in women. The optimal cut-off points of 32 cm and 37 cm for men and women, which similar to Chinese populations. Therefore, it can be used as a cost-effective tool to predict NAFLD.

**Trial Registration**: Thai Clinical Trials Registry (TCTR20210329006)

## Introduction

Evidence from several recent studies has shown the potential of neck circumference (NC) as an anthropometric measure that is well reflective for human health [1, 2]. Neck circumference is a surrogate for measuring upper body subcutaneous fat deposition, a predictor of metabolic syndrome (MetS), and a promising predictor of Non-Alcoholic Fatty Liver Disease (NAFLD) [3–5]. Compared to waist circumference as the surrogate for central obesity, NC measurement is easy and highly reproducible with little variation [6]. Its value is less likely influenced by age or associated with increasing age [7]. In addition, recent studies have also demonstrated that upper body subcutaneous adipose tissue has a stronger relationship with metabolic disorders than visceral adipose tissue (VAT) or central obesity [8].

Nonalcoholic fatty liver disease has now become the leading cause of chronic liver diseases worldwide [9]. As it strongly correlates with insulin resistance (IR), NAFLD is considered a risk factor for future development of metabolic syndrome and its complications such as type 2 diabetes mellitus and cardiovascular diseases [10]. Therefore, early detection and efficacious intervention at its primary stage will enhance prognosis and prevent such complications. At present, the most used method for NAFLD diagnosis is ultrasonography, which is not easy to organize and not cost-effective at community levels. Finding simpler and more effective indicators for population NAFLD screening is therefore an urgent need.

Several studies have examined the potential of neck circumference as the predictor of NAFLD in the adult population including four studies from the People Republic of China [4, 6, 11, 12] and one each from India [13] and Iran [14]. Three studies from China reported the optimal cut-off NC of 37.25 to 38.5 cm for male adults with the area under the curve (AUC) of 0.71–0.768, the sensitivity of 55.22 to 79.5% and specificity of 59.1 to 71%; and 32.9 to 34.5 cm for female adults with the AUC of 0.649–0.798, sensitivity of 62–79% and specificity of 67–77%. Iran’s study reported a slightly higher optimal cut-off of 39.25 cm for males (AUC 0.821, sensitivity 79% specificity 69%) and 34.85 cm for females (AUC 0.807, sensitivity 84%, specificity 64%). A study in India, among adults with pre-diabetes, reported a more gender-comparable optimal cut-off of 36.25 cm for males (AUC 0.551, sensitivity 72%, specificity 40%) and 35.25 cm for females (AUC 0.662, sensitivity 80%, specificity 62.7%). For the neck circumference to be a public health utility as a predictor of NAFLD, it should be widely applicable across global populations with comparable optimal cut-off among populations with similar race/ethnic backgrounds. In addition, Asian ethnicities are more susceptible to metabolic syndrome (MetS), type 2 diabetes and NAFLD than Europeans for equivalent levels of over-nutrition, because of body composition, particularly adiposity and muscle bulk difference [15].

In this study, we examined the potential of neck circumference in predicting NAFLD among the adult population in Thailand. Specifically, we aimed to: (a) examine the association of NC with NAFLD prevalence, and (b) determine the AUC, optimal cut-off, sensitivity, and specificity of neck circumference in identifying the presence of NAFLD and liver fibrosis (LF) among the employees of an academic institution in Bangkok, Thailand. We hypothesized that: (a) neck circumference can be used as a valid and independent predictor of NAFLD, and (b) the optimal cut-offs for both genders were similar to those reported in the Chinese population due to Thais sharing a similar Mongoloid ethnic background.

## Materials and methods

### Study subjects

This cross-sectional study was a part of the CU 100 years: A healthy liver and heart campaign—Hepatitis B, Hepatitis C, Cardiovascular risk and Non-alcoholic fatty liver disease (NAFLD) among staff members at Chulalongkorn University and King Chulalongkorn Memorial Hospital Bangkok, Thailand project. The project recruited 766 employees of an academic institution in Bangkok who participated in the annual physical check-up during 2019–2020.

Inclusion criteria were any staff currently working at the Chulalongkorn University or King Chulalongkorn Memorial Hospital who was older than 40 years. Participants with neck malformation or surgery history, thyroid dysfunction that may interfere with NC measurement, and those who were pregnant were excluded from the study.

Sample size calculation was conducted post-hoc based on the following formula [16]: n_control_ = (Z ^2^_α/2_ P (1-P)/d ^2^ and n_total_ = n_control_/(1-prevalence), where n_control_ = number of non NAFLD, n_total_ = number of total subjects, P = expected specificity (0.71 for men and 0.77 for women) [4], d = allowable error (0.05), Z_α/2_ = standard values for type I error at an α level of 0.05 (1.96), and prevalence = prevalence of NAFLD among Thai adults (0.18 for men and 0.23 for women) [17]. This resulted in the required sample size of 388 for men and 353 for women. Our study subjects of 133 men and 502 women therefore indicated that the statistical power was limited for men and satisfactory for women.

**Fig 1:**
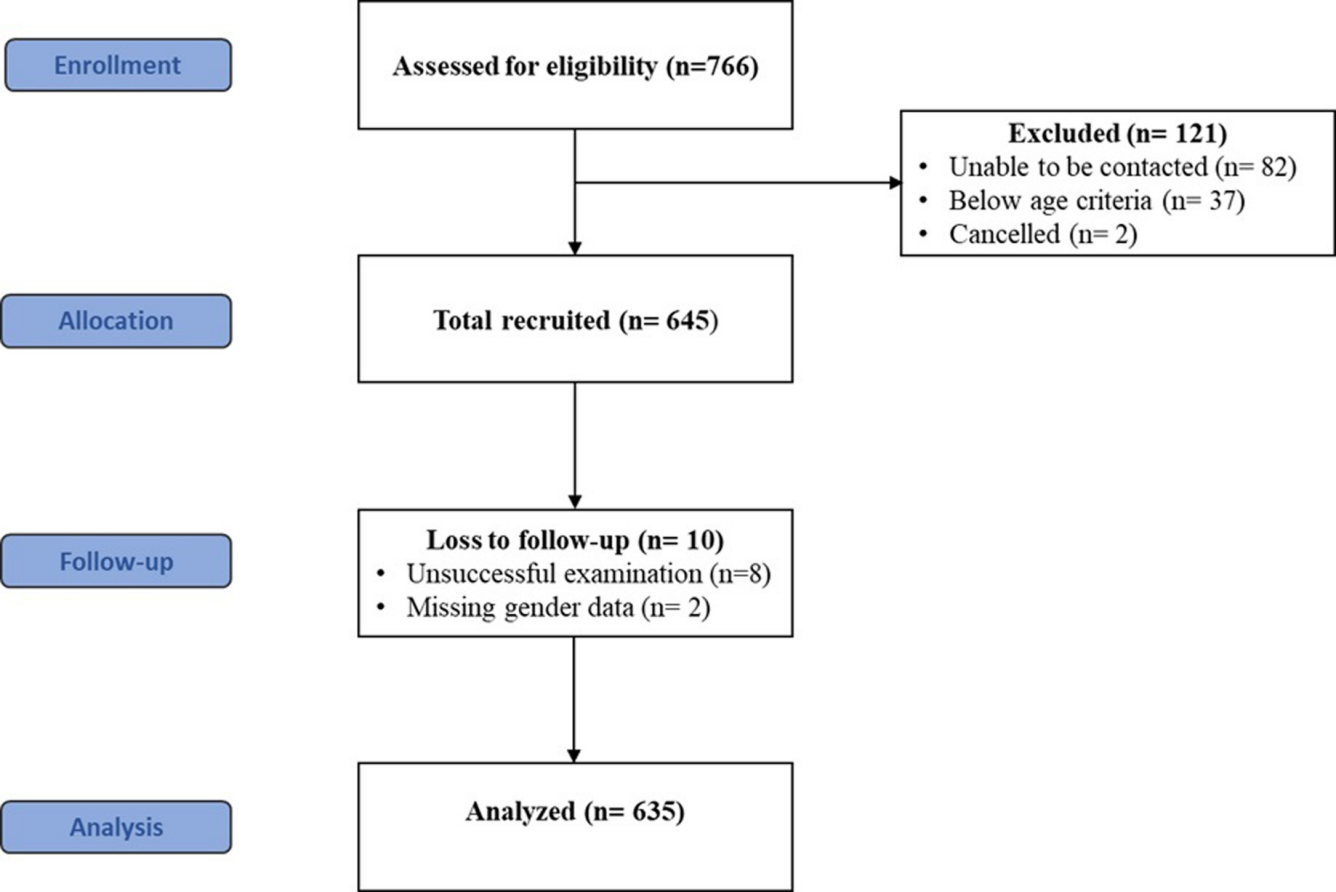
CONSORT flow diagram.

A total 766 were recruited, 82 not contactable, 2 cancelled, 37 were below the age criteria, leaving 645 in the examination. However, eight were unsuccessfully examined, two were missing on gender, leaving 635 participants in the final analysis (Fig 1). The project was approved by the Ethics Committee of Chulalongkorn University Faculty of Medicine (IRB no. 546/59), and all subjects provided informed consent.

### Anthropometric measurements

Anthropometric parameters, including weight (kg), height (cm), waist circumference (WC, cm) and hip circumference (HC, cm) were measured on the check-up day. Participants were requested to dress in light attire and were bare footed for weight measurement. Body mass index (BMI) was calculated by dividing body weight in kilograms by the square of the participant’s height in meters (kg/m^2^). Waist circumference was measured horizontally to the nearest 1.0 mm using a standard measuring tape at a point right above the iliac crest on the mid-axillary line at minimal respiration. Hip circumference was measured in centimeters, at the level of maximum extension of the hip, with leg close together. Waist to Hip Ration (WHR) was calculated as the ratio between WC and HC. Blood pressure was examined after 10 minutes rest in a sitting position on the upper left arm, and the systolic (SBP) and diastolic blood pressure (DBP) were recorded. Neck circumference was assessed around the inferior margin of the laryngeal prominence and perpendicular to the long axis of the neck, to the nearest 1.0 mm. by trained nurses. In men, NC was measured just below the laryngeal prominence, while participants standing upright and the head in the horizontal plane position with shoulders down [18].

### Biochemical assays

Biochemical indicators including fasting blood glucose (FBG), total cholesterol (TC), triglycerides (TG), Low-density lipoprotein (LDL), high-density lipoprotein (HDL), aspartate aminotransferase (AST), and alanine aminotransferase (ALT) were obtained by the annual health check-up. On the day of check-up, fasting blood samples were taken from a vein puncture then it was immediately centrifuged (3,000 rpm) for 10 min at 4°C, and the serum was separated for further analysis. Fasting plasma glucose, TC, high-density lipoprotein-cholesterol (HDL-c), and TG were analyzed using the enzymatic method, while low-density lipoprotein-cholesterol (LDL-c) was estimated using the Friedewald equation (LDL-c = [total cholesterol]—[HDL-c]—[TG]/5). Hepatic steatosis index (HSI) was calculated as: 8 x (ALT/ aspartate aminotransferase ratio) + body mass index (+2, if female; +2, if diabetes mellitus) [19].

### Nonalcoholic Fatty Liver Disease (NAFLD) and liver fibrosis assessment

Transient elastography was done with the FibroScan (Echosens, Paris, France) medical device, and the controlled attenuation parameter (CAP) was used to detect and quantify liver steatosis, while the liver stiffness measurement (LSM) was selected as the parameter to quantitate liver fibrosis [20]. After an overnight fast, the examination was performed using an M probe on the right lobe of the liver through intercostals spaces with the participants in dorsal decubitus with the right arm in maximal abduction by experienced technologists unaware of clinical data. In case of BMI >30 kg/m^2^ or unreliable measurement from the M probe, the XL probe was used. Ten successful measurements were performed on each participant, and only cases with 10 successful acquisitions were taken into account for this study [21].

All scans were performed by the same investigator. The cut-off value for the diagnosis of liver steatosis was a CAP value >238 dB.m-1, while the cut-off value for the diagnosis of significant liver fibrosis (F2) was liver stiffness >7 kPa [22]. The cut-off value for the diagnosis of liver steatosis (NAFLD) was a CAP value >238 dB.m-1

There were several missing data including: 1 to 3 participants (0.16–0.47%) for age, SBP and DBP, cigarette smoking and alcohol drinking statuses; and 97 to 106 participants (15.28 to 16.69%) for FBS, TC, TG, HDL and LDL cholesterol, AST and ALT levels. Missing data for continuous variables were managed by cold-deck imputation where gender-specific mean values were used to assign to those participants for whom data were missing. Missing data for categorical data were managed by random overall imputation where a participant was selected at random from the total of participants, and the value for that person was assigned to all those participants for whom the information was missing [23].

### Statistical analysis

All analyses were conducted separately for male and female participants. Continuous data were summarized and presented by mean ($\bar{x}$) and standard deviation (SD) in case of normal distribution, or by median and interquartile range (IQR) in case of skew distribution. Categorical data were summarized and presented by frequency and percentage. Group comparison between those with versus without NAFLD was conducted by unpaired t-test or Chi-square test (or Fisher’s Exact where appropriate) respectively for continuous and categorical data.

All analyses were conducted separately for male and female participants. Continuous data were summarized and presented by mean ($\bar{x}$) and standard deviation (SD) in case of normal distribution, or by median and interquartile range (IQR) in case of skew distribution. Categorical data were summarized and presented by frequency and percentage. Group comparison between those with versus without NAFLD was conducted by unpaired t-test or Chi-square test (or Fisher’s Exact where appropriate) respectively for continuous and categorical data.

Associations of neck circumference with the probability of NAFLD was then examined by logistic regression analyses using odds ratio (OR) and the corresponding 95% confidence interval (CI) as measure of association. Possibility for non-linearity of neck circumference and log odds NAFLD association was addressed by transforming neck circumference using a restricted cubic spline with three knots. Such transformation however did not reveal the evidence of non-linearity (as inferred from no significant different among the slopes of splines, detail not shown), the normal term of neck circumference was therefore used in the logistic regression analyses. Potential confounding effects of age, personal histories of HT, T2DM, dyslipidemia, cigarette smoking and alcohol consumption; BMI, waist circumference, SBP, DBP, FBS, TC, TG, LDL, HDL, AST, and ALT were taken into account by analyzing model 2. In MODEL I, all potential confounders were put into the model. In MODEL II, backward stepwise selection procedure was conducted and only the variables with the association p-value of <0.1 were left in the final model.

Performance of neck circumference in identifying NAFLD began by examining the distribution of observed and predicted NAFLD probabilities across the level of neck circumference. Then it was evaluated both in term of calibration and discrimination. Calibration was evaluated by using the Hosmer-Lemeshow goodness of fit test as well as observed proportions versus predicted probabilities. The intercept approaching zero and the slope approaching one reflects a perfect calibrated model [24].

Discrimination was evaluated by plotting the receiver operating characteristic (ROC) curves, and AUC with 95% confidence interval (CI) were calculated to explore the discriminative ability of neck circumference as diagnostic tests for detecting NAFLD and determine optimal sex-specific NC cutoffs in relation to NAFLD. The AUC of 0.5 was interpreted as “no discrimination”, 0.7 to 0.8 as “acceptable”, 0.8 to 0.9 as “excellent”, and more than 0.9 as “outstanding” [25].

The Youden index was used to determine the optimal cutoff points [26]. The STATA Version 15 [27] for Windows was used to perform all data analyses, and the statistical significance level was set at 0.05.

## Results

### Comparison between NAFLD and non-FLD participants

Of the 133 men and 502 women, 88 and 232 had NAFLD with the prevalence of 66.17 and 46.22% respectively. Concerning LF, 6 men and 12 women had such conditions, with the prevalence of 4.51 and 2.39% respectively.

Group comparison between participants with and without NAFLD showed that neck circumferences were significantly larger among those with NAFLD, with the mean (SD) of 38.53 (0.31) versus 35.83 (0.48) centimeters for men (p < .0001) and 33.58 (0.24) versus 31.08 (0.14) centimeters for women (p < .0001) (Table 1). Those with NAFLD also were older, and had higher values of anthropometric measures (weight and BMI, WC, HC, neck to height ratio and waist to hip ratio), blood pressure (SBP and DBP), fasting blood glucose, and liver function tests (AST and ALT). They had higher levels of triglyceride and lower levels of HDL cholesterol. NAFLD had higher personal histories of HT and T2DM than those without NAFLD, particularly among women. In addition, men, and women with NAFLD has a significantly higher value of HSI.

**Table 1:** Comparison between participants with and without NAFLD, stratified by gender.

Variables	Women (n = 502)					Men (n = 133)				
	NAFLD (n = 232)		Non_FLD (n = 270)		*p-value*	NAFLD (n = 88)		Non_FLD (n = 45)		*p-value*
	Mean	(SD)	Mean	(SD)		Mean	(SD)	Mean	(SD)	
Age (years)	52.64	(0.45)	50.41	(0.54)	0.0014	49.62	(0.76)	49.78	(1.00)	0.903
Height (cm)	157.04	(0.32)	156.99	(0.35)	0.9083	169.34	(0.63)	168.09	(0.88)	0.2496
Weight (kg)	65.91	(0.70)	59.14	(3.35)	0.0345	77.34	(1.44)	64.54	(1.20)	<0.001
Neck circumference (cm)	33.58	(0.24)	31.08	(0.14)	<0.001	38.53	(0.31)	35.83	(0.48)	<0.001
Waist circumference (cm)	87.21	(0.61)	76.53	(0.60)	<0.001	93.88	(1.35)	83.06	(1.34)	<0.001
Hip circumference (cm)	100.91	(0.52)	94.03	(0.47)	<0.001	101.59	(0.87)	95.31	(0.87)	<0.001
Body mass index (kg/m^2^)	26.70	(0.26)	24.15	(1.51)	<0.001	26.98	(0.50)	22.88	(0.45)	<0.001
Neck to height ratio	0.214	(0.002)	0.198	(0.001)	<0.001	0.228	(0.002)	0.213	(0.003)	0.0001
Waist to hip ratio	0.863	(0.003)	0.813	(0.004)	<0.001	0.922	(0.008)	0.871	(0.011)	0.0004
Systolic blood pressure (mm Hg)	128.59	(1.04)	117.31	(1.07)	<0.001	131.77	(1.40)	122.84	(2.02)	0.0004
Diastolic blood pressure (mm Hg)	81.23	(0.63)	76.37	(0.70)	<0.001	84.48	(0.98)	81.73	(1.41)	0.1072
Platelet count ((× 10^3^/μL))	297.97	(3.94)	277.94	(3.95)	0.0004	262.24	(4.74)	244.83	(6.53)	0.0336
Mean corpuscular volume (fL)‡	87.60	(10.40)	88.20	(10.15)	0.5887	87.90	(8.70)	88.00	(7.60)	0.3929
Fasting blood glucose (mg/dL)	98.42	(1.60)	90.39	(1.04)	0.0001	101.08	(3.05)	95.58	(4.19)	0.2929
Total cholesterol (mg/dL)	220.46	(2.12)	219.88	(2.50)	0.8582	213.70	(3.78)	209.70	(7.93)	0.6059
Triglycerides (mg/dL)	119.54	(3.80)	92.36	(2.61)	<0.001	143.96	(7.94)	98.40	(7.16)	0.0003
LDL cholesterol (mg/dL)	139.14	(1.85)	137.86	(2.20)	0.6539	136.03	(3.61)	132.80	(7.64)	0.6648
HDL cholesterol (mg/dL)	57.41	(0.70)	63.55	(0.86)	<0.001	48.88	(1.11)	57.22	(2.35)	0.0004
Aspartate aminotransferase (U/L)	23.37	(0.97)	19.90	(0.38)	0.0017	24.20	(0.80)	23.18	(1.00)	0.4419
Alanine aminotransferase (U/L)	23.30	(0.97)	19.83	(0.38)	0.0018	25.21	(0.83)	23.97	(1.06)	0.3708
ALT/AST ratio‡	1	(0)	1	(0)	0.7597	1	(0)	1	(0)	0.7750
Cigarette smoking status†	11	(4.07)	4	(1.72)	0.188	12	(13.64)	6	(13.33)	/1.00
Alcohol drinking status†	28	(10.37)	37	(15.95)	0.082	32	(36.36)	14	(31.11)	0.570
Personal history of hypertension†	57	(21.11)	25	(10.78)	0.002	11	(12.5)	4	(8.89)	0.733
Personal history of diabetes†	33	(12.22)	8	(3.45)	0/0	9	(10.23)	6	(13.33)	0.576
Personal history of dyslipidemia†	57	(21.11)	41	(17.67)	0.367	11	(12.50)	4	(8.89)	0.773
Significant Liver stiffness (>7 kPa)†	1	(0.43)	11	(4.07)	0.008	5	(5.68)	1	(2.22)	0.663
Hepatic steatosis index (HSI)	36.93	(0.27)	32.79	(0.23)	0.0001	34.91	(0.53)	30.84	(0.47)	0.0001
HBS Ag positive†	7	(2.59)	6	(2.59)	1.000	3	(3.41)	3	(6.67)	0.406
Anti HCV positive†	1	(0.37)	2	(0.86)	0.598	0	(0)	1	(2.22)	0.338

† number (%)

‡median (interquartile range or IQR)  ALT/AST ratio = Aspartate aminotransferase/ Alanine aminotransferase ratio.

HBS Ag = Hepatitis B virus surface antigen  Anti HCV = Antibody against hepatitis C virus.

### Association between metabolic syndrome parameters and NAFLD

Metabolic syndrome parameters including BMI, WC, SBP, DBP, FBG, TG, and HDL were associated with liver steatosis among women. For men, however, no correlation of FBG, DBP and liver steatosis observed possibly due to its limited sample size. In addition, HSI showed a strong correlation with liver steatosis in both men and women (Table 2).

**Table 2:** Correlation between the controlled attenuation parameter (CAP) quantifying liver steatosis and metabolic syndrome parameters, stratified by gender.

Parameters	Women			Men		
	*r*	(95%CI)	*p-value*	*r*	(95%CI)	*p-value*
Body mass index (kg/m^2^)	0.55	(0.49, 0.61)	*0*.*001*	0.51	(0.38, 0.63)	*0*.*001*
Neck circumference (cm)	0.43	(0.36, 0.50)	*0*.*001*	0.46	(0.31, 0.58)	*0*.*001*
Waist circumference (cm)	0.57	(0.51, 0.63)	*0*.*001*	0.53	(0.40, 0.65)	*0*.*001*
Systolic blood pressure (mm Hg)	0.38	(0.30, 0.45)	*0*.*001*	0.29	(0.13, 0.44)	*0*.*001*
Diastolic blood pressure (mm Hg)	0.30	(0.22, 0.38)	*0*.*001*	0.14	(-0.03, 0.30)	*0*.*1125*
Fasting blood glucose (mg/dL)	0.25	(0.17, 0.33)	*0*.*001*	0.12	(-0.05, 0.29)	*0*.*1671*
Total cholesterol (mg/dL)	0.05	(-0.04, 0.14)	*0*.*6809*	-0.05	(-0.22, 0.12)	*0*.*5758*
Triglycerides (mg/dL)	0.31	(0.23, 0.39)	*0*.*001*	0.33	(0.17, 0.48)	*0*.*001*
LDL cholesterol (mg/dL)	0.08	(-0.01, 0.17)	*0*.*4013*	-0.08	(-0.25, 0.09)	*0*.*3337*
HDL cholesterol (mg/dL)	-0.33	(-0.40, -0.25)	*0*.*001*	-0.25	(-0.41, -0.09)	*0*.*004*
Hepatic steatosis index (HSI)	0.56	(0.49, 0.61)	*0*.*001*	0.49	(0.35, 0.61)	*0*.*001*

r = Pearson’s correlation coefficient  CI = Confidence interval.

### Association between neck circumference and NAFLD

Logistic regression analyses show that neck circumference was significantly and independently associated with NAFLD among women (Table 3), with the OR (95%CI) of 1.17 (1.05, 1.32) for the best fitted model (Model II, as inferred from the Hosmer-Lemeshow goodness of fit test result). For men, however, neck circumference was significantly associated with NAFLD only in the unadjusted (or crude) analytical result, possibly due to its small sample size.

**Table 3:** Association between neck circumference and NAFLD, stratified by gender.

MODEL	Women				Men			
	OR	(95%CI)	*p-value*	*GOF*	OR	(95%CI)	*p-value*	*GOF*
Crude Model	1.50	(1.37, 1.65)	*0*.*001*	*0*.*0011*	1.39	(1.19, 1.63)	*0*.*001*	*0*.*001*
MODEL I	1.16	(1.03, 1.31)	*0*.*014*	*0*.*0231*	1.12	(0.86, 1.46)	*0*.*389*	*0*.*0398*
MODEL II	1.17	(1.05, 1.32)	*0*.*006*	*0*.*2817*	1.07	(0.87, 1.32)	*0*.*527*	*0*.*3062*

GOF = Hosmer-Lemeshow goodness of fit test (p-value)  OR = Odds ratio.

MODEL I: adjusted for age; personal histories of hypertension, diabetes and dyslipidemia; cigarette smoking and alcohol drinking status; body mass index, waist circumference, systolic and diastolic blood pressures, fasting blood glucose, total cholesterol, triglyceride, HDL and LDL cholesterol, aspartate aminotransferase and alanine aminotransferase levels.

MODEL II: adjusted for age; cigarette smoking and alcohol drinking status; waist circumference, systolic blood pressure, and fasting blood glucose levels; these variables were obtained by a backward stepwise selection procedure, leaving those with p< 0.1 in the final model.

**Fig 2:**
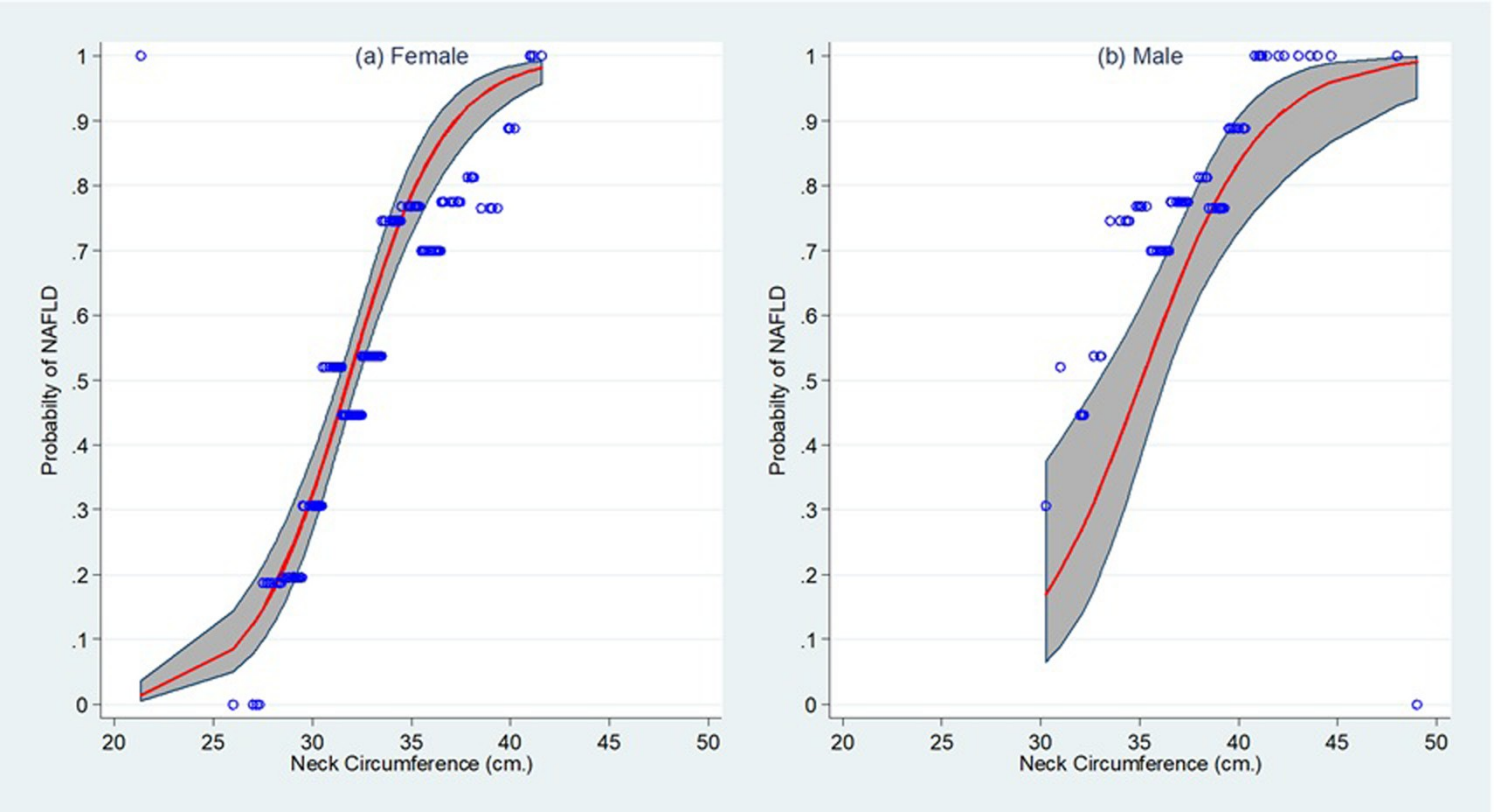
Distribution of the observed and predicted probabilities of having non-alcoholic fatty liver disease (NAFLD) across neck circumference level among for (a) female and (b) male participants. Thick line represents the expected probability, hollow circle represents the observed probability, shade represents the 95% confidence interval of the expected probability.

Plotting of the NAFLD probability by neck circumference level showed that NAFLD probabilities increased progressively according to neck circumference level, with the lean s-shape pattern of association in both women and men (Fig 2).

**Fig 3:**
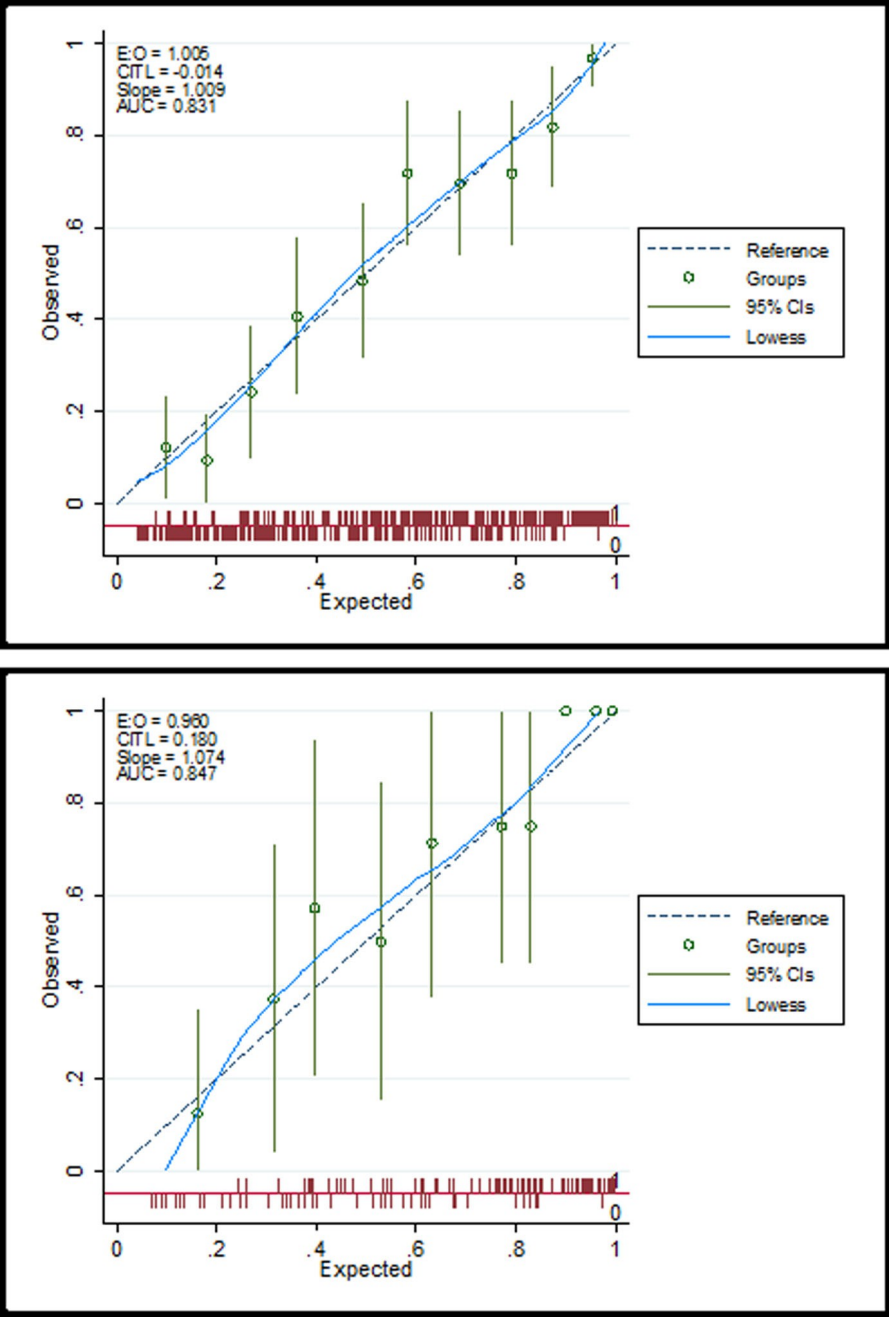
Calibration plot for observed and predicted probabilities of NAFLD (neck circumference as the independent variable) among (a) Female and (b) male participants. AUC = area under the receiver operating characteristic (ROC) curve; CI = confidence interval; CITL = calibration in the large; E:O = Expected:Observed.

Distribution patterns of both the observed and expected NAFLD probabilities were well consistent with each other, particularly for women. This was confirmed by the calibration plots (Fig 3), the results of which revealed negligible departure from zero of the calibration in the large (CITL) values and trivial departure from one for the slopes. The expected NAFLD probability for women was slightly less than the observed probability, while it was contrary for men.

Concerning the accuracy of neck circumference to discriminate between those with from those without NAFLD, the AUC results were between 0.7 to 0.8 and thus indicated this was acceptable, with the suggested optimal cut-offs of 32.07 and 37.07 cm respectively for women and men (Table 4). The corresponding sensitivity and specificity values were 70.70 and 62.10% respectively for women, and 70.50 and 68.90% for men.

**Table 4:** Predictive validity of neck circumference in identifying NAFLD, stratified by gender.

Parameter	Women		Men	
	Mean	(95%CI)	Mean	(95%CI)
AUC	0.757	(0.719, 0.795)	0.759	(0.673, 0.846)
Cut-off	32.07	(31.62, 32.51)	37.07	(36.20, 37.93)
Sensitivity (%)	70.70	(64.90, 76.10)	70.50	(59.80, 79.70)
Specificity (%)	62.10	(55.50, 68.30)	68.90	(53.40, 81.80)
PPV (%)	68.50	(62.70, 73.90)	81.60	(71.00, 89.50)
NPV (%)	64.60	(57.90, 70.80)	54.40	(40.70, 67.60)
LR+	1.86	(1.56, 2.24)	2.26	(1.44, 3.57)
LR-	0.47	(0.38, 0.58)	0.43	(0.29, 0.63)
Odds ratio	3.96	(2.73, 5.74)	5.28	(2.44, 11.44)

AUC = area under the receiver operating characteristic (ROC) curve; CI = confidence interval; LR+ = likelihood ratio for positive results; LR- = likelihood ratio for negative results; NPV = negative predictive value; PPV = positive predictive value.

## Discussion

The relationships between NAFLD and NC, metabolic and anthropometric measurements were evaluated in this study. As the prevalence of NAFLD is high and increasing, its clinical and economic burden will become tremendous [9]. As NAFLD is considered the liver indicator of metabolic syndrome [28] and the most common indicator for liver transplantation [29]; simple tools for the early detection of NAFLD are therefore imperative as the first step in the endeavor to ameliorate its enormous public health impact, especially in resource limited settings. In this study we demonstrated that NC could be used as one of such tools. In addition, NC is the most discriminative indicator that can predict the development of NAFLD. Its discriminative ability to differentiate between those with and without NAFLD was acceptable, as indicated by the AUC of approximately 0.75. In addition, HSI also provide good estimates of development of NAFLD. The use of HSI is also a reasonable indicator for NAFLD prediction.

This study identified optimal cut-offs for NC of about 32 cm for women and 37 cm for men, which was comparable to those identified for Chinese adults sharing a similar ethnic background to the Thai population. That may also support the generalizability of using NC as an appropriate screening tool in the early steps for NAFLD detection. Previous studies indicated that NC reflects the upper-body fat deposition and contributes to the proportion of systemic free fatty acids (FFA) which are involved in pathogenesis of NAFLD [30]. Our evidence was strong for women, while this was not the case for men, possibly due to the limited sample size. Similarly, previous studies demonstrated that NC has a highest predictive value to predict NAFLD in women, whereas WC has a highest predictive value in men [12].

Our findings showed a high NAFLD prevalence of 66.17% and 46.22% respectively for men and women aged greater than 40 years. The high prevalence of MetS was also recognized in the current study. This might be because of a high prevalence of overweight and obesity in both men and women who participated in the study. As reported, the prevalence of obesity in Thailand keeps increasing from 33.9% in 2012 to 44.8% in 2018 even in rural areas [31]. With a sedentary lifestyle of our participants, the obesity prevalence is higher than expected.

In addition, age and obesity are significant risk factors of NAFLD. Therefore, the high prevalence of NAFLD observed in this study might be related to a high prevalence of obesity and age of the study populations. As a previous study indicated, NC reflects abdominal fat accumulation and obesity, it is therefore related to fatty liver and hepatitis [32]. In addition, it was demonstrated that NC significantly correlates with plasminogen activator inhibitor 1, and leads to inflammation of the liver and cardiovascular disease [33, 34].

In this study, weight, BMI, NC, WC, WHR, FBS, triglycerides, ALT, AST were significantly higher whereas HDL was significantly lower among participants with NAFLD compared to those without NAFLD, which was in accordance with a previous study [35]. Additionally, a strong relationship between MetS parameters and the prevalence of NAFLD existed in both genders. Among women participants, NAFLD prevalence was higher in participants who reported hypertension and diabetes. Similarly, a previous study also reported that NAFLD prevalence was two-fold higher in hypertensive individuals than in non-hypertensive individuals [36]. Meanwhile, diabetes shows a directional relationship with NAFLD since diabetes increases the risk of NAFLD and NAFLD worsens hyperglycemia in diabetic patients [37, 38]. This could attribute to the role of intrahepatic fat in the development of insulin resistance and MetS. In NAFLD, excessive fat accumulation in the liver increases the mobilization of free fatty acids (FFAs) and consequently promotes insulin resistance [39]. Elevated reflux FFAs can also reduce adiponectin secretion thus stimulating triglycerides and VLDL synthesis in the hepatocytes. In addition, FFAs overruns in the pancreas may cause lipotoxicity and lead to β-cell dysfunction and hyperglycemia [39].

The limitations of the study were that our participants were employees of the hospital and university in the center of Bangkok, thus the results from this study could not be extrapolated to the Thai population countrywide. A regional multi-center study is recommended for future study. In addition, this study corrected blood indicators from a routine check-up which was approximately three months before elastography and the NC examination. Moreover, liver biopsy, which is the gold standard of liver steatosis quantification, or magnetic resonance imaging (MRI)-based modality might be better indicators to diagnose patients with NAFLD than the performance of CAP. However, in a recent meta-analysis, it has been shown that CAP correlates well with the amount of steatosis assessed by liver biopsy. Additionally, the etiology of liver disease, including HBV and HCV, fibrosis staging, and diabetes are not associated with the discrepancies between CAP and histological assessment of liver steatosis [40].

## Conclusion

This study concluded that NC is associated with NAFLD prevalence and can be used as a strong and accurate measurement to predict a prognosis of NAFLD and MetS in a cost-effective manner. The optimal cut-offs for NC to identifying the presence of NAFLD are 32 cm and 37 cm for women and men, respectively. Those NC cut-offs for NAFLD prediction are almost similar to those reported among Chinese populations. Also, NAFLD had significantly higher metabolic markers including age, weight, BMI, NC, WC, WHR, FBS, triglycerides but HDL was significantly lower among participants with NAFLD compared to those without NAFLD.

## Supporting information

S1 Table Association between hepatic steatosis index (HIS) and NAFLD, stratified by gender. (DOCX)

S1 Fig Distribution of the observed and predicted probabilities of having non-alcoholic fatty liver disease (NAFLD) across hepatic steatosis index (HSI) level among for (a) female and (b) male participants. Thick line represents the expected probability, hollow circle represents the observed probability, shade represents the 95% confidence interval of the expected probability.
(DOCX)

S1 File Dataset of the study. (XLS)
