# Supplementary material for: Neck circumference as a screening measure for identifying NAFLD among a group of academic employees in Bangkok, Thailand
Source: PLoS One. 2022 Feb 17;17(2):e0263826. doi: 10.1371/journal.pone.0263826 (PMC8853466; doi:10.1371/journal.pone.0263826)
Supplement: S1 Table — (DOCX) [file pone.0263826.s001.docx]

**Supporting information**

Logistic regression analyses show that HSI was significantly associated with NAFLD only in the unadjusted (or crude) analytical result. In crude model, p-value of GOF for women and men of 0.1269 and 0.1147, respectively, it was concluded that this model fit the observed data.

**Table 1.** Association between hepatic steatosis index (HIS) and NAFLD, stratified by gender

| **MODEL** | **Women** | | | |  | **Men** | | | |
| --- | --- | --- | --- | --- | --- | --- | --- | --- | --- |
|  | **OR** | **(95%CI)** | ***p-value*** | ***GOF*** |  | **OR** | **(95%CI)** | ***p-value*** | ***GOF*** |
| Crude Model | 1.31 | (1.24, 1.39) | *0.001* | *0.1269* |  | 1.37 | (1.19, 1.58) | *0.001* | *0.1147* |
| MODEL I | 0.59 | (0.17, 2.09) | *0.414* | *0.01* |  | 0.50 | (0.14, 1.76) | *0.283* | *0.001* |
| MODEL II | 1.09 | (0.99, 1.21) | *0.068* | *0.1414* |  | 1.10 | (0.87, 1.40) | *0.398* | *0.4835* |

GOF= Hosmer-Lemeshow goodness of fit test (p-value) OR=Odds ratio

MODEL I: adjusted for age; personal histories of hypertension, diabetes and dyslipidemia; cigarette smoking and alcoholic drinking status; body mass index, waist circumference, systolic and diastolic blood pressures, fasting blood glucose, total cholesterol, triglyceride, HDL and LDL cholesterol, aspartate aminotransferase and alanine aminotransferase levels.

MODEL II: adjusted for age; cigarette smoking and alcoholic drinking status; waist circumference, systolic blood pressure, and fasting blood glucose levels; these variables were obtained by backward stepwise selection procedure, leaving those with p<.1 in the final model.
