# Supplementary material for: Neck circumference as a screening measure for identifying NAFLD among a group of academic employees in Bangkok, Thailand
Source: PLoS One. 2022 Feb 17;17(2):e0263826. doi: 10.1371/journal.pone.0263826 (PMC8853466; doi:10.1371/journal.pone.0263826)
Supplement: S1 Fig — Distribution of the observed and predicted probabilities of having non-alcoholic fatty liver disease (NAFLD) across hepatic steatosis index (HSI) level among for (a) female and (b) male participants. Thick line represents the expected probability, hollow circle represents the observed probability, shade represents the 95% confidence interval of the expected probability. (DOCX) [file pone.0263826.s002.docx]

**Figure 1** Distribution of the observed and predicted probabilities of having non-alcoholic fatty liver disease (NAFLD) across hepatic steatosis index (HSI) level among for (a) female and (b) male participants. Thick line represents the expected probability, hollow circle represents the observed probability, shade represents the 95% confidence interval of the expected probability.

Plotting of the NAFLD probability by HIS showed that HSI showed a probability to predict NAFLD in both women and men (Supplementary Figure 1). Based on figure 1, the probability of NAFLD increases as the HSI score increases. The greater the HSI score the probability of experiencing NAFLD also increases.
